# Validation of a Brief CES-D Scale for Measuring Depression and Its Associated Predictors among Adolescents in Chi Linh, Hai Duong, Vietnam

**DOI:** 10.3934/publichealth.2016.3.448

**Published:** 2016-07-04

**Authors:** Nguyen Duc Thanh, Bui Tu Quyen, Truong Quang Tien

**Affiliations:** 1 Department of Hospital Management, Health Management Training Institute, Hanoi School of Public Health, 138 Giang Vo, Hanoi, Vietnam; 2 Department of Biostatistics, Faculty of Fundamental Science, Hanoi School of Public Health, 138 Giang Vo, Hanoi, Vietnam; 3 Department of Health Education, Faculty of Social Sciences, Behavior and Health Education, Hanoi School of Public Health, 138 Giang Vo, Hanoi, Vietnam

**Keywords:** depression, validity, reliability, associated factors, adolescent and youth

## Abstract

**Objectives:**

The aims of this paper were to confirm the validity and reliability of a brief CES-D measure for depression and identify the associated factors with the depression among adolescents and youth in Chi Linh, Hai Duong province, Vietnam.

**Methods:**

We used data from a prospective-longitudinal study of adolescents and youth (aged 13–17 at baseline) and their parent (N = 1402 mother/father-child dyads). Adolescents' depression was assessed in 2009 and 2013. Parents' connectedness was measured in 2013. Confirmatory factor Analysis (CFA) was used to certify the elementary factors produced by PCA using Comparative Fit Index (CFI), Tucker Lewis Index (TLI) and RMSEA. Multivariate linear regression was used to predict the factors associated with depression.

**Results:**

The results demonstrate that the depression items correspond as CFI (0.89), TLI (0.87) and RMSEA (0.084) are acceptable fit indices. The mean score of depression among adolescents and youth was 30.9 (Min = 16 and Max = 70; SD = 8.3). Age (*β* = −0.01; CI 95% = −0.1; −0.03), parent and youth can talk freely (*β* = −0.042; CI 95% = −0.08; −0.001) and good health status (*β* = −0.07; CI 95% = −0.1; −0.03) were found to be significantly associated with depression.

**Conclusions:**

Depression scale should be widely applied for screening the depression symptoms of adolescents and youth population. The necessary strategies should be implemented to improve the adolescent and young population's depression status.

## 1. Introduction

Depression is one of the mental health problems which is considered as an important aspect of the youth and adolescent health. The disease is closely related to a range of behaviours such as the suicide attempt and suicidal behaviour among different population groups regardless of their sex and age [1].

Apart from that, the depression symptoms could also affect the people's normal life in the future [2]. Several factors have also been found to be associated with depression. The most serious symptoms of depression were found in youths aged 15 to 17 years and associated significantly with female youths, lower socioeconomic status, poor interpersonnal relations and chronic health conditions [3]. Furthermore, lack of social support and poor sleep hygiene were found to be significantly associated with depression at undergraduate students [4]. High school connectedness, high school record to be a protective factor of depression of high school students [5],[6]. A study has recently found that parents' emotional connectedness was significantly associated with depression in adolescents [7]. Apart from these associated factors with depression, little has been known about the association between the parents' connectedness with adolescent activities and their depression, especially in Vietnam. Given that adolescent's depression is associated with the above mentioned short- and long-term consequences, there is an urgent need to understand the status of depression and its associated factors among adolescence.

The Center for Epidemiologic Studies Depression Scale (CES-D) was developed to diagnose of depression, to identify the depressive symptoms and risk of having disorder of a person [8]. The CES-D consists of 20 items such as “You felt that you were just as good as other prople” and “You were happy”, takes four-point Likert scale, ranging from: “rarely or none of the time” with 0 point to “most or all of the times” with three points. The total score of scale is from 0 to 60, in which the higher score shows the serious depressive status. The CES-D has shown good validity and reliability for depression evaluation in the different population groups [1],[9],[10]. For instance, the CES-D was helpful in identifying the depressive symptoms in rural Chinese residents who attempted to have suicidal behaviours and shown good construct validity and internal consistency [1].

Although being popular, the CES-D is still concerned in terms of its factor structure and detail items [11],[12]. The studies validating the CES-D scale have given various recommendations in specific population groups when comparing to the original scale, which consists of four factors (depressed, somatic, interpersonal and positive). Several studies from China and Vietnam have demonstrated that the four-factor model of the CES-D greatly fitted [1],[9]. When conducting the research with CES-D, it is important to examine whether the scale is reliable and valid for the study population, because of different ethnic and socio-demographic groups may have different factor structures [13]. A study applying the CES-D among the Chinese suicidal attempters have shown that two-factor and three-factor models were identified aside from the original four-factor model [1],[14],[15]. The two-factor model of depression (positive and negative affect) has been identified at a study of the adolescents health in Chi Linh town, Hai Duong province, Vietnam [16],[17]. In this study, we want to confirm the validity and reliability of this depression scale and identify the associated predictors with depression among the adolescents in Chi Linh town, Hai Duong province, Vietnam.

## 2. Methods

**Figure 1.**
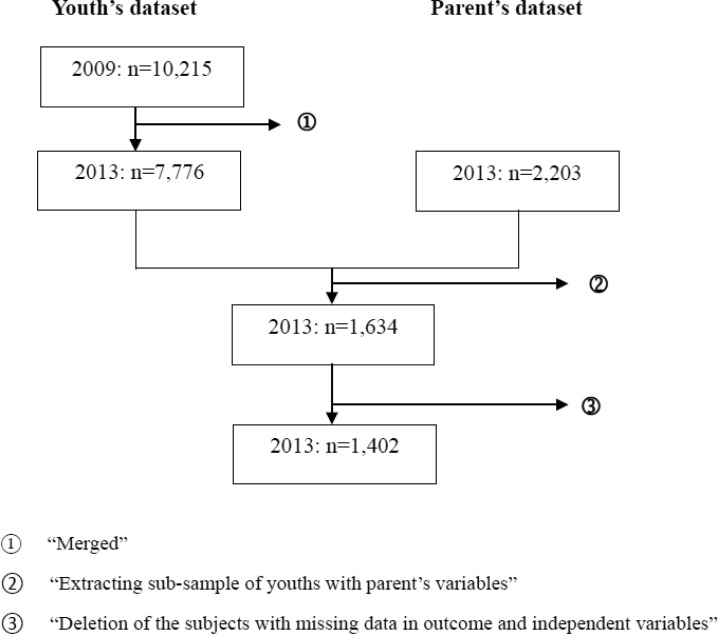
Derivation of study samples. Figure 1 shows the derivation of study samples from each dataset (i.e. youth and parent). After applying the exclusion criteria, the study samples comprised 1,402 observations for the analyses).

A sample of 10,215 adolescents aged from 13 to 17 was studied in 2009. In 2013, these adolescents were repeatedly studied, the sample size was 7,776. The data of their depression status and other individual variables were collected in both periods of time. The data of their parents' connectedness to adolescent activities were also collected in 2013 and merged with the adolescent data in 2013. After merging, 1402 valid cases were analyzed (Figure 1).

The trained interviewers were responsible for collecting the data of these studies. The self-reported questionnaire was provided to the appropriate adolescents to collect data of depression while face-to-face interviews were done with their parents to collect the data of connectedness. Written informant consents were obtained from each subject prior to the interview. The parents were asked for the permission of their under 18 aged children's participation in the study. All the interviews were conducted at the subjects' homes and without an appearance of a third person. Each interview lasted about an hour in duration.

Apart from adolescent personal socio-demographic data such as gender, age, education level, residence, the other data were collected. The adolescent parents' connectedness such as parents and youth can talk freely, parents care about children's emotion were also collected. The scale variable of *“parent and youth can talk openly”* was developed by combining five individual variables such as *“you often talk to your children about youth and adolescent topic (puberty, gender, sex,…)”*, *“your children often tell you about their private problems (studying, loving, health…)”*, *“your children ask you all the things when they don't know/understand”*, *“you can talk to your children about the problems in life without shy”* and *“your children can talk to you about their worries/sad without shy”*. This scale variable was then recoded into two values: Yes and No. Similarly, the scale variable of *“parent cares about their child's emotions”* was developed by combining three individual variables such as *“parent knows what their children are concerned or worried about”*, *“parent knows their children's emotions”* and *“parent consider their children's problems as their own”*. This scale variable was also then recoded into two values: Yes and No. In this study, the modified CES-D comprises 16 items as a brief CES-D scale was used because it was cross-cultural adapted [17]. The cross-cultural adaptation included a translation in standard language plus adjustment of cultural words, idoms and context [18]. A modified CES-D takes five-point Likert scales, ranging from 1 corresponding “never” to 5 corresponding “always”. The total score of scale is from 16 to 80, in which the higher scores the more depressive symptoms adolescent and youth reveal.

Data were analysed with SPSS 16 and Stata 12. Reliability was evaluated by internal consistency (Cronbach's alpha). If the alpha value is 0.7 or higher, this suggests that the test is reliable and the scale could be used properly [18],[19]. Structural Equation Model (SEM) was used as an analysis in the construction of depression model. Confirmatory factor Analysis (CFA) was conducted with the collected data in 2013 to certify the elementary factors produced by Exploratory Factor Analysis (EFA) with the collected data in 2009 using Comparative Fit Index (CFI), Tucker Lewis Index (TLI) and RMSEA. These indices' criteria must be met for a satisfactory fit model: (1) Chi-square/df ratio should be between 1 and 5; (2) CFI and TLI must approach 1 [21],[22]; (3) the Standardized Root Mean Square Residual (SRMR) must be less than 0.1; (4) RMSEA should be up to 0.09 with 90% confidence interval values below 0.1 [22]. A multivariate linear regression was used to predict the factors associated with depression.

The study was approved by the Ethics Committee of Hanoi School of Public Health, Vietnam. The studied individuals signed the informed consent form.

## 3. Results

### 3.1. Demographic characteristics of the study sample

Among 1402 youths, 49.8% were male and 50.2% were female. The mean of their age was 21.7 ± 2. There was 47% youth living in urban area, about 90% of youth belong to the Kinh group while the rest from other ethnic minority groups. Nearly one fifth of youth lived in poor family, the proportion of youth being good/very good health status was 82% (Table 1).

**Table 1.** Background characteristics of Youth in 2013.

Characteristics		Total (N = 1402)		Male (n = 698; 49.79%)		Female (n = 704; 50.21%)	
Mean of age in years (SD)		21.7	(2.0)	21.7	(2.0)	21.7	(2.0)
**Commune**							
Sao Do		304	(21.7)	144	(20.6)	160	(22.7)
An Lac		151	(10.8)	77	(11.0)	74	(10.5)
Pha Lai		288	(20.5)	165	(23.6)	123	(17.5)
Van An		218	(15.6)	103	(14.8)	115	(16.3)
Le Loi		269	(19.2)	131	(18.8)	138	(19.6)
Ben Tam		67	(4.8)	29	(4.2)	38	(5.4)
Hoang Tien		105	(7.5)	49	(7.0)	56	(8.0)
**Area**							
Urban		659	(47.0)	338	(48.4)	321	(45.6)
Rural		743	(53.0)	360	(51.6)	383	(54.4)
**Ethnicity**							
Kinh		1259	(89.8)	624	(89.4)	635	(90.2)
Non-Kinh		143	(10.2)	74	(10.6)	69	(9.8)
**Health status**							
Very good		158	(11.3)	112	(16.1)	46	(6.5)
Good		993	(70.8)	486	(69.6)	507	(72.0)
Average		240	(17.1)	95	(13.6)	145	(20.6)
Poor		11	(0.8)	5	(0.7)	6	(0.9)

### 3.2. Reliability of the depression scale

Table 2 shows that the total score of youth's depression in 2013 were 30.9 ± 8.3; the lowest score was 16 and the highest score was 70. There were 2 factors, negative and positive affects, which are made up from 16 items. Cronbach's Alpha was 0.84 (0.87 for negative affect and 0.75 for positive affect) also proved the quite good correlation among items of depression.

**Table 2.** Means, standard deviations, and internal consistencies of the depression scale and subscales in 2013.

Depression scale	Mean (SD)	Min	Max	Cronbach's alpha
Total score	30.9 (8.3)	16	70	0.84
**Factors**				
Negative affect	25.1	13	65	0.87
Positive affect	10.6	3	15	0.75

### 3.3. Validity of the depression scale

Table 3 shows the factor loadings from EFA and CFA. The dataset in 2009 was used for EFA. Several conditions must be carried out to test whether the items were suitable in order to run the analysis. One of the tests was Barlett's Test of Sphericity and Kaiser-Meyer-Olkin Test (KMO). Barlett's Test of Sphericity showed significant value of .000, indicating *p* < 0.05. Thus, it means that the correlation among items is sufficient to run the factor analysis. In KMO, the value is 0.93 (greater than 0.5). This means that these items are relevant to the factor analysis performed and showed no serious multicollinearity data. After Barlett's Test of Sphericity and Kaiser-Meyer-Olkin Test were performed, the Rotating Matrix Component table was used to test the construct validity of each question constructed. It was found that there were two components that exist after Varimax rotation. All factor loadings were 0.6, so they all were acceptable. Cronbach's Alpha was 0.87 for factor 1 – “Negative affect” and 0.72 for factor 2 – “Positive affect”.

**Table 3.** Validity and reliability of a brief CES-D measure for depression using exploratory factor analysis (EFA) and confirmatiory factor analysis (CFA).

			EFA for sample in 2009*		CFA for sample 2013*	
**Factor 1: Negative affect**						
1	were bothered by things that get used to		0.62*		0.59	
2	do not like to eat		0.60		0.60	
3	felt could not shake off the blue		0.62		0.69	
5	had trouble keeping your mind on what you were doing		0.66		0.69	
6	felt stress		0.67		0.70	
7	felt too tired to work		0.67		0.60	
9	thought the life had been failure		0.64		0.62	
10	felt fearful		0.65		0.66	
12	talked less than usual		0.63		0.65	
13	felt lonely		0.73		0.75	
14	people were unfriendly to you		0.69		0.60	
15	felt sad		0.77		0.72	
16	felt people disliked you		0.67		0.69	
**Factor 2: Positive affect**						
4	felt good as others		0.70		0.64	
8	hope to have a bright future		0.74		0.81	
11	were happy		0.79		0.67	
Cronbach's Alpha						
	Factor 1: Negative affect		0.87		–	
	Factor 2: Positive affect		0.72		–	
KMO (Kaiser-Meyer-Olkin) Measure of Sampling Adequacy			0.93			
Barlett's Test of Sphericity						
*Approx. Chi Square*			51435.4			
*df*			120			
*Sig.*			0.000			
RMSEA (Root mean squared error of approximation)			–		0.084	
CFI			–		0.89	
TLI (Tucker-Lewis index)			–		0.87	
Chi-Square			–		5767.44	
p value			–		< 0.001	

* Factor loadings

The CFA was the last analysis of the dataset in 2013 used to verify the basic factors that have been produced by the EFA and to validate these constructs. Before CFA can run, several specifications have to conducte on the distribution of normality, multicollinearity, the sample size and the scale of measurement. These requirements were all met in this survey. Testing of depression constructs showed the Chi Square Goodness-of-Fit χ2 = 5767, with *p* < 0.001 and other specifications such as RMSEA value (0.084), CFI value (0.89) and TLI value (0.87) were acceptable fit indices.

### 3.4. Associated factors of depression

Table 4 presents the crude coefficients (*Univariate linear regression model*) and adjusted coefficients (*Multivariate linear regression model*) for log of depression score (outcome) in 2013. The crude model revealed that age, residence, parent and youth can talk openly, and youth's health statuses were related with youth's depression (*p* < 0.05).

**Table 4.** Univariate and multivariate analysis of characteristics associated with depression among youth/adolescents in 2013 (n = 1402).

Characteristics		Mean (SD) of Log(depression score)	Univariate linear regression model: *β* (CI95%)		Multivariate linear regression model: *β* (CI95%)
(1)		(2)	(3)		(4)
**Age**		–	−0.007* (−0.01; −0.001)		−0.01* (−0.10; −0.03)
**Gender**					
Male		3.39 (0.26)	–		
Female		3.41 (0.27)	0.02 (−0.01; 0.05)		0.02 (−0.01; 0.04)
**Residence**					
Urban		3.41 (0.26)	–		
Rural		3.38 (0.26)	−0.03* (−0.06; −0.01)		−0.03 (−0.05; 0.001)
**Parent and youth can talk openly**					
No		3.40 (0.26)	–		
Yes		3.37 (0.26)	−0.03* (−0.07; −0.004)		−0.042* (−0.08; −0.001)
**Parent care about their child's emotions**					
No		3.42 (0.26)	–		
Yes		3.39 (0.26)	−0.02 (−0.06; 0.02)		−0.038 (−0.08; 0.01)
**Health status**					
Other (normal/not good)		3.45 (0.27)	–		
Good		3.39 (0.26)	−0.07* (−0.1; −0.03)		−0.07** (−0.1; −0.03)

*: *p* < 0.05; **: *p* < 0.01.

The multivariate linear regression analysis controlling for the simultaneous association of predict factors revealed age, parent and youth can talk openly, and good health status remained significant (*p* < 0.05). The depression score will decrease by approximately 1% when age increases by one year (*p* < 0.05). The depression score will be 4% lower for youths who can talk openly with their parents than for those who cannot (*p* < 0.05). The score also will be 7% lower for youths who have good health status than for those who have normal or not good health status (*p* < 0.001). There were no relationship between gender, residence, parent care about their child's emotions and youth's depression score.

## 4. Discussion

The purposes of this analysis were to examine the brief version CES-D scale (16 items) in an adolescent and youth population in terms of its reliability and validity, and then explore the associated factors of depression among this target population. Screening for depression and understanding its influential factors are primary important steps in overall depression management and prevention from this problem proactively.

### 4.1. Reliability of the depression scale

The analysis in this paper assessed the reliability and validity of the 16-item depression scale among sample of 1402 youths in Chi Linh town. The CES-D scale has been shown to be a reliable measure for assessing depressive symptoms of general population and especially in adolescent population with high internal consistency [1],[8],[9],[23]. This scale is actual CES-D scale of Radloff (1977) without four items 11, 16, 17 and 20 among 20 item questions of the original one [8]. There was no confirmed reason for removing those items from the original items from the CES-D scale as a result of previous study. In the literature, some studies used the brief revised version of the CES-D scale for some specific target population as adolescents or older adults, and proved their effectiveness [24],[25].

The finding indicates that the 16-item scale has satisfactory reliability in depression assessment at Chi Linh in 2009 with Cronbach's Alpha was 0.84. Evaluation of the structure underlying response to positive feeling and negative feeling demonstrated reasonable construct validity for each subscale, suggesting that the 16-item version CES-D is a useful and valid instrument for depressive symptoms in adolescent and youth population. Such result of depression scale reliability is consistent with the findings of the previous studies of Nguyen Thanh Huong among adolescents in 2004 and in 2005 in Viet Nam with Cronbach's Alpha of 20-item scale are 0.87 and 0.85 respectively [9]. So far, we have not found the study report in which the 16-item depression scale among Vietnamese adolescents was presented, and therefore, it is a limitation for the comparison.

### 4.2. Validity of the depression scale

The validity of the 16 items depression scale was confirmed by examining the outcome of CFA. CFA was utilized to determine the items to measure the constructs of depression. Through the CFA analysis, these indicator variables representing the aspects of depression as positive or negative feelings were proven. Testing for depression scale construct showed the Chi-square test with significance (*p* < 0.001); and all necessary indicators such as CFI = 0.89; TLI = 0.87; and RMSEA = 0.084) met the criteria for an acceptably valid scale to measure the depression status [19],[21].

This shows the regression model proposed by the study fits the data. This indicates that the scale can be applied to measure the depression status among specific population like as adolescent and youth. In fact, this scale applied to estimate the depression status of adolescent and youth in Chi Linh town. This is eligible in terms of the reliability and validity. The 16-item scale of screening depression should be used to measure the depression status among other target groups of population.

### 4.3. Depression status and associated factors

This analysis would like to present factors, which are associated with depression status. Understanding these factors deeply helps the health promotion planner to suggest the appropriate solution to address the depression matters among target subjects.

Many studies showed that some demographic characteristics, forms of child maltreatments associated significantly with the depression status. Adolescents who live in urban area are more likely to have better physical health, higher self-esteem, and less depression. Those live in families with the poor family connectedness or the quality of relationship between children and other members of the family are more likely to get depression. Huong's findings emphasized that experienced maltreatment collectively are significant association with depression [26],[27]. Yuen (2006) also stressed the significant relationship between the experienced emotional maltreatment and depression status among Malaysian adolescent population [27].

According to Hailemariam's study in Ethiopia population (2012), there were many risk factors of depression such as age, marital status, chronic non-communicable disease status, alcohol consumption [28]. In this analysis, the crude models revealed that age, gender, residence, parent and youth can talk openly and youth's health status were related with depression among adolescent and youth. However, the multivariate analysis by linear regression controlling for the confounding factors, revealed only age, parent and youth can talk openly and health status remained to be significant predicted factors of adolescent and youth's depression status. The more growing up the lower score of depression status the youth gets. Adolescents and Youths who can talk openly with their parents, and had good health status were more likely avoiding the depression problem. These findings are consistent with conclusions in Huong (2006) in Vietnamese adolescents [26] and Choo (2006) in Malaysian adolescents [27]. The scale variable of *“parent and youth can talk openly”* consisted of five mentioned individual variables. They focus on the frequency of communication between parents and adolescents; about adolescent related topics, on the easiness of sharing the sensitive or private issues, sadness or worries, as well as life skills. The mental health programs should be developed focusing on these aspects. In this study, there was no relationship between parent care about child's emotions and adolescent and youth's depression. This is contrary with the study conducted in European area in 2015 [7]. This difference may be due to cultural differences about the relationship between parents and their children in two different locations.

This study has several strengths. First, the sample was relatively large and chosen randomly. Therefore, most of analyses had reasonable power. Second, the tool was developed based on the standardized questions so that it was likely valid and reliable. Third, the interviewers were experienced and strictly trained. The data collection process was also supervised frequently. Thus, the collected data were reliable. The last meaningful strength was that the study likely provided the valid and reliable tool for screening the depression problem among adolescents and youths in Vietnam.

Some limitations of this study should be noted. The bias of most concern in this study is the recall bias because the data were collected by self-reported questionnaires. The depressive symptoms are answered by recall instead of using a method of diagnostic interview. The cross-sectional data analysis for examining the associated predictors is also difficult to explain the actual influential factors. The result of linear regression analysis indicates the structural model of the scale was gender invariance. Therefore, the identified structure of CES-D in this study may not be equivalent for adolescent boys and girls. This issue should be further examined in other studies of this topic.

## 5. Conclusions

The results of the analysis proved that the 16-item depression scale suited for applying to estimate this problem among adolescents and youth population. Age, parent and youth communication and health status found to be remarkable associated factors for depression status. The study findings indicate the importance of strategies to integrate mental health services into primary health care at local level aiming to promote the mental health status. Local policy makers and practical public health professionals should consider these findings and recommendations in the design of mental health promotion program for adolescents and youth in Chi Linh community.
